# The Value of Liquid Biopsies for Guiding Therapy Decisions in Non-small Cell Lung Cancer

**DOI:** 10.3389/fonc.2019.00129

**Published:** 2019-03-05

**Authors:** Jatta Saarenheimo, Natalja Eigeliene, Heidi Andersen, Marja Tiirola, Antti Jekunen

**Affiliations:** ^1^Department of Pathology, Vasa Central Hospital, Vaasa, Finland; ^2^Department of Biological and Environmental Science, Nano Science Center, University of Jyväskylä, Jyväskylä, Finland; ^3^Department of Oncology, Vasa Central Hospital, Vaasa, Finland; ^4^Department of Oncology and Radiotherapy, University of Turku, Turku, Finland; ^5^Department of Pulmonology, Vasa Central Hospital, Vaasa, Finland

**Keywords:** ctDNA, NSCLC, EGFR, personalized therapy, cancer, precision medicine

## Abstract

Targeted therapies have allowed for an individualized treatment approach in non-small-cell lung cancer (NSCLC). The initial therapeutic decisions and success of targeted therapy depend on genetic identification of personal tumor profiles. Tissue biopsy is the gold standard for molecular analysis, but non-invasive or minimally invasive liquid biopsy methods are also now used in clinical practice, allowing for later monitoring and optimization of the cancer treatment. The inclusion of liquid biopsy in the management of NSCLC provides strong evidence on early treatment response, which becomes a basis for determining disease progression and the need for changes in treatment. Liquid biopsies can drive the decision making for treatment strategies to achieve better patient outcomes. Cell-free DNA and circulating tumor cells obtained from the blood are promising markers for determining patient status. They may improve cancer treatments, allow for better treatment control, enable early interventions, and change decision making from reactive actions toward more predictive early interventions. This review aimed to present current knowledge on and the usefulness of liquid biopsy studies in NSCLC from the perspective of how it has allowed individualized treatments according to gene profiling and how the method may alter the treatment decisions in the future.

## Introduction

Lung cancer is the most common cancer worldwide, accounting for 11.6% of all cases of cancer, and is the leading cause of cancer-related mortality (1). The World Health Organization classifies lung cancer into two major types based on its biology, therapy, and prognosis as non-small-cell cancer (NSCLC) and small-cell lung cancer (SCLC), with NSCLC being the more common type as it accounts for >80% of all lung cancer cases (2). The identification and advanced understanding of molecular abnormalities in lung cancer has made it possible to define specific molecular driver mutations for the disease subsets, and several biomarkers have emerged as predictive and prognostic markers for NSCLC, impacting the selection of treatment. Testing these gene alterations is important for the identification of efficacious therapies and avoidance of therapies that are unlikely to provide clinical benefit. The gold standard for molecular analysis has been tissue biopsy, but new liquid biopsy methods with cell free DNA (cfDNA) are rapidly introduced in clinical practice, providing new possibilities to optimize the treatments.

Plasma and serum includes variable amounts of molecular signatures originating from the tumor, and the process for detecting these molecular signatures in blood samples is called liquid biopsy. Tumor information can be obtained from circulating tumor DNA (ctDNA), circulating tumor cells (CTC), exosomes, platelets, and microRNAs. The ctDNA is released through a lysis of apoptotic and necrotic cells or digestion of tumor cells by macrophages or by direct secretion of DNA by tumor cells (3). ctDNA is a subset of total cfDNA and varies between 0.01 and 90% (4), depending on tumor stage, vascularization, burden, biological features such as apoptotic rate and metastatic potential of the cancer cells, and the factors affecting the patient's blood volume (5). The half-life of ctDNA in the blood stream varies between 16 min to 2.5 h, making ctDNA a “real time” biomarker reflecting the tumor burden (4, 6). CTCs are tumor cells that are detached from the solid tumor mass and disseminated in the blood circulation. To detach cancer cells from the primary tumor, the cells need to undergo the cellular process of epithelial-mesenchymal transition (7), which allows tumor cells to gain motility and migratory capacity, resulting in their penetration into the blood stream, where they circulate as CTCs (8). CTCs participate in tumor metastasis, as it is believed that metastasis is initiated by a sub-group of CTCs seen in the blood (9). ctDNA and CTCs are the most widely investigated markers in liquid biopsies of patients with cancer. In addition to blood, promising results of liquid biopsies have been obtained from other body liquids, such as saliva and urine (10–12).

Less than 20% of patients with lung cancer undergo surgery, which limits the size of available tissue samples for small biopsies and cytological analysis. In some cases, treatment decisions need to be made without any tissue verification, as biopsy samples are not always available due to health limitations of the patients because patients with lung cancer often have severe symptoms of chronic obstructive pulmonary disease (13, 14). This makes the role of liquid biopsies more pronounced. Liquid biopsies have notable advantages over tissue biopsies as they provide information on the complete heterogeneity (both spatial and temporal), sampling procedures are minimally invasive or totally non-invasive, and repeated sampling is possible for following up treatment efficacy, development of resistance, and cancer progression. Moreover, liquid biopsy is less expensive, and the sample preparation is faster. However, the scarcity of gene alterations requires highly sensitive methods to achieve reliable results and avoid false-negative results. Research on liquid biopsy methods has been growing rapidly, allowing for the development of several new suitable methods. However, sensitivity has remained a key obstacle in the development of these methods. Sensitivity needs to be as low as 0.01% because more than 50% of the known alterations are present at allelic frequency of <0.25% (15). Fortunately, there are methods that can reach this sensitivity level, where the lowest sensitivity that can be reached is 0.01%, from where the error rate of DNA polymerase starts to be the limiting factor (16). In addition, analytical specificity impacts the reliability of results, which is generally >95% (17). The sample collection and pre-treatment conditions also affect the results; thus, plasma collection in stabilization solution or EDTA tube is widely recommended (18). The currently available methods can detect single nucleotide variations, indels, deletions, copy number variations, and rearrangements at a sensitivity level of approximately 0.01–2% (17, 19, 20).

Despite the advantages of liquid biopsy, there are still limitations that need to be addressed before liquid biopsy-based detection, monitoring, and treatment guidance can be fully applied in the management of lung cancer. These include its limited availability and high cost. Moreover, the sensitivity and reliability vary between different types of liquid biopsies and between tissue and liquid biopsies. Some studies reported high concordance between tissue biopsies and liquid biopsies (21, 22) in terms of EGFR sensitizing mutations. By contrast, broader analyses of 45 somatic mutations in tissue biopsy found that only 66% of these mutations were detected in liquid biopsy specimens via NGS (23). Also, a comparison between two different methods of liquid biopsy showed that the less sensitive methods yielded false-negative T790M results (24). Aside from method-related limitations, the detection of mutations can also depend on the type of malignancy. Some cancer types shed significant amounts of tissue while others do not, and the amount of shed tissue can also vary in one tumor type (25). The amount of ctDNA also depends on the stage of the disease. Patients with no detectable ctDNA have small volume disease, lepidic growth pattern, mucinous tumors, or isolated leptomeningeal disease (26). Subclones carrying driver mutations are also more prone to release DNA (27). This means that positive ctDNA results can be regarded as true positive, while negative results are not completely accurate and should be verified with repeated analysis or with tissue biopsy analysis if possible. Moreover, deep sequencing and most sensitive methods may yield false-positive results when various mutations are targeted, such as RAS or TP53 genes. These findings are clinically significant as they can predict worse survival or resistance to available targeted therapies (28, 29), but they may also lead to mischaracterization of a concomitant tumor.

In this paper, we aimed to review the current state of liquid biopsies as a method for guiding clinical decision making in NSCLC. As the ctDNA is the most commonly used biologic element of liquid biopsies, this review primarily focuses on ctDNA, but CTCs are also included. The primary focus of this review is on how liquid biopsies have changed the individualized treatment approach, how it is predicted to influence treatment decisions, and how the different mutations are introduced in practice from the perspective of therapy decisions.

## PubMed and Clinical Trial Search

To investigate the rapid development and the role of liquid biopsy in treatment decisions, we searched PubMed for article published in 2010–2018 (until September 1, 2018). The first search used the keyword “NSCLC cancer,” which yielded 40,659 articles. Further, when “liquid biopsy” was added in the search, the number of articles decreased to 247. These articles were further reviewed, and the articles in which liquid biopsy methods were used to guide treatment decisions (15 articles) were included in the review and are summarized in Table 1. The review showed increasing interest in liquid biopsy; in 2015, only 0.32% of the articles reported on liquid biopsy, while in 2018 1.6% of published articles reported it (publication numbers are presented in Supplementary Table 1).

**Table 1:** Published articles in PubMed in which liquid biopsy methods are used to guide treatment decisions.

**Alteration**	**Patients**	**Platform**	**Result**	**References**
EGFR	1	NGS	A rare triple EGFR mutation R670W/H835L/L833V was detected, and the patient responded well on second generation TKIs	(30)
Multiple targets	116	Guardant 360	Comprehensive cfDNA testing impacted clinical decisions in 1/4 to 1/3 of initial and subsequent lines of treatment in advanced NSCLC patients. Responses based on cfDNA are durable and change treatment decisions at initial presentation and at progression	(31)
EGFR	52	ARMS	In the Liquid group, 3 of 4 patients with discordant results between tumor and liquid biopsy showed treatment responses favoring the liquid biopsy	(32)
ALK	1	ARMS	An EML4-ALK rearrangement was found after acquired resistance to EGFR TKI treatment. Crizotinib was administered. The patient's lung lesions continued to progress after 1 month of crizotinib treatment, and pemetrexed-bevacizumab was initiated. After two cycles of chemotherapy, the metastatic cancers shrunk, and the patient maintained stable disease at his last follow-up	(33)
Multiple targets	50	dPCR	Patients with both TP53 and EGFR mutations before treatment had worse overall survival than those with only EGFR Patients who progressed without T790M had worse PFS during TKI continuation and developed alternative alterations, including small-cell lung cancer-associated copy number changes and TP53 mutations. Longitudinal plasma analysis can help identify dominant resistance mechanisms, including non-druggable genetic information that may guide clinical management.	(28)
EGFR	119	ddPCR	Plasma genotyping using digital polymerase chain reaction was clinically useful for the selection of patients who had progressed during first-line EGFR-TKI therapy for treatment with osimertinib	(34)
ALK	1	NGS	GCC2-ALK was identified and functionally validated as a constitutively activated fusion in NSCLC. The patient benefited from crizotinib treatment initially and then ceritinib after progression, suggesting GCC2-ALK as a novel therapeutic target for ALK inhibitors	(35)
EGFR	1,026	RT-PCR	Large-scale EGFR testing in the blood of unselected advanced NSCLC patients is feasible and can be used to select patients for targeted therapy when testing cannot be done in tissue. The characteristics and clinical outcomes to TKI treatment of the EGFR-mutated patients identified are undistinguishable from those positive in tumor.	(15)
EGFR	3		Three cases are presented that were successfully treated with osimertinib after progression on 1st and 2nd generation EGFR TKIs. The presence of T790M mutation was detected from ctDNA of the patients	(36)
EGFR	1		Without histological analysis, the origin of the primary ocular metastasis was uncertain. In this context, a LB showing an activating mutation in EGFR and circulating tumor cells positive for TTF1 led to the diagnosis of NSCLC and targeted therapy	(37)
EGFR	18	dPCR	Monitoring levels of EGFR mutation in plasma allows resolving doubts that frequently arise in daily clinical practice and constitutes a major step toward achieving personalized medicine	(12)
ALK	1	FoundationACT	Here we report an ALK+ NSCLC patient who had disease progression after ceritinib and then alectinib where an ALK G1202R mutation was detected on ctDNA prior to enrollment onto a trial of another next generation ALK inhibitor, lorlatinib. The patient's central nervous system (CNS) metastases responded to lorlatinib together with clearance of ALK G1202R mutation by repeat ctDNA assay. However, the patient developed a new large pericardial effusion. Resected pericardium from the pericardial window revealed SCLC transformation with positive immunostaining for synaptophysin, chromogranin, and ALK (D5F3 antibody)	(38)
EGFR	48	NGS	The ctDNA T790M mutation was detected in 50% of NSCLC patients. Among assessable patients, osimertinib gave a partial response rate of 62.5% and a stable disease rate of 37.5%	(39, 40)
Multiple targets	68	NGS	Over 80% of patients had detectable ctDNA, concordance between paired tissue and blood for truncal oncogenic drivers was high and patients with biomarkers identified in plasma had PFS in the expected range	(26)
EGFR	1		A novel urine ctDNA assay was utilized and confirmed T790M positive status. The patient was started on a third generation TKI, which led to a measurable clinical response	(41)

The search was conducted on September 1, 2018 using the keyword “NSCLC liquid biopsy.”

A more detailed review of the 247 articles showed that majority (32%) of the articles were review papers (Supplementary Table 2). When only clinical trials were included, only eight articles remained (Supplementary Table 2). From these eight articles, seven reported on epidermal growth factor receptor (EGFR) mutations. More precisely, three articles were about the T790M mutation, treatment with osimertinib, and the potential of liquid biopsies for detecting the mutation and to guide tyrosine kinase inhibitor (TKI) therapy (39, 40, 42). One clinical trial assessed the changes in ctDNA EGFR mutation status within 1 day (43). Two of the articles concentrated on the CTC-based methods in NSCLC genetic assessment (27, 44). The seventh article was about EGFR and aimed to determine the clinical relevance of urinary cfDNA as an alternative source of liquid biopsy tumor biomarker (45). Finally, the last article reported on the evaluation of methylation markers in plasma DNA for lung cancer detection and discrimination of malignant from non-malignant lung disease (46).

Physicians treating lung cancer are eager to use new drugs and treatment strategies in clinical practice to improve patient outcomes. Thus, there will be no hesitation to use also new modalities for controlling treatments and to help treatment decision making. Several research studies are currently conducted to produce reliable evidence on the clinical use of liquid biopsy. According to data from clinicaltrials.gov, there are many studies, both on-going and conducted, on lung cancer and liquid biopsies that will be published soon. The search results are summarized and presented in Supplementary Table 3. Surprisingly, most of the studies concentrate on CTCs, and only few are based on cfDNA or ctDNA analysis, while the current published papers and guidelines are mainly based on ctDNA (Supplementary Table 3). The high interest on CTCs may imply that in the future, CTCs may have a bigger role in lung cancer detection, treatment decision, and follow-up than it does currently. This may lead to an ultra-sensitive method to detect and culture circulating CTCs in blood or in cellblocks, which could further be used for immunohistochemistry analysis of the lung cancer biomarkers p40, TTF1, ALK, ROS1, and PD-L1.

## Current Role of Liquid Biopsies in Deciding the Treatment for Lung Cancer

The US Food and Drug Administration (FDA) and the European Medical Association have approved the use of and published guidelines on the modalities for detecting different EGFR mutations, including liquid biopsy specimens, to monitor and decide the appropriateness of TKI treatment for patients with mutations (47). In addition, the latest National Comprehensive Cancer Network (NCCN) guidelines for NSCLC provide principles for molecular and biomarker analysis, and numerous gene alterations impacting therapy selection have been identified (available at NCCN.org). The genetic composition of each patient is investigated either via tissue biopsy or liquid biopsy or both before treatment decision. While assessing these alterations to select efficacious targeted therapies is crucial, it can be equally important to avoid therapies that are unlikely to provide clinical benefit.

### Epidermal Growth Factor Receptor (EGFR)

Sensitizing EGFR mutations are among the predictive biomarkers for NSCLC, and majority of previous research and clinical trials have focused on EGFR mutations [i.e., (48–51)]. The most commonly found EGFR mutations in NSCLC are deletions in exon 19 and point mutation in exon 21 (p.L858R). Both mutations result in activation of the tyrosine kinase domain and are associated with sensitivity to the small molecule TKIs, such as erlotinib, gefitinib, and afatinib (52). These sensitizing mutations are found in approximately 50% of Asian and 10% of Caucasian patients with NSCLC (53). Also, other less commonly found alterations, such as exon 19 insertions, and point mutations at exon 21 (L861Q, S768I) and exon 18 (G719X), have been shown to be sensitive to EGFR TKI therapy [(54–56). In addition, deep sequencing has revealed new rare mutations which may benefit of TKIS, such as rare triple EGFR mutation R670W/H835L/L833V (30). However, some cell clones with EGFR mutations lack responsiveness to EGFR TKI therapy, including most of exon 20 insertions, and their incidence is a predictive factor for resistance to clinically achievable efficacy of TKIs (57). Moreover, primary resistance to TKI therapy is associated with ALK and ROS1 rearrangements and KRAS mutations. In addition, approximately 50% of NSCLC patients with EGFR mutants treated with EGFR TKIs will develop acquired resistance to the T790M mutation (58). Acquired resistance may also be associated with the histologic transformation from NSCLC to SCLC (59).

The FDA has approved the use of liquid biopsy for the analysis of both sensitizing mutations and resistance mutations, and results where liquid biopsies are used to guide therapy decisions are strongly supporting this (15, 32). The progression free survival times and overall survival times are at same level as they are compared to studies where therapy decisions are made based on tissue biopsy. Moreover, in situations where the histological analysis are not possible can the diagnosis and therapy decisions rely on ctDNA and CTC based methods (37). In addition, sensitizing EGFR mutations, particularly the abundance and fluctuations of EGFR genes, have been used as basis for determining the appropriate first-line therapy. Treatment response has been monitored by observing the abundance of known driver mutations, where the fluctuations of EGFR-mutant abundance in the serial plasma cfDNA samples are in accordance with the changes in tumor size assessed via imaging scans (60). In addition, longitudinal EGFR mutation levels in plasma correlated with the tumor response determined using RECIST criteria (12). In 19 patients treated with afatinib for ≥24 weeks, the number of EGFR mutant alleles detected in cfDNA via digital polymerase chain reaction (PCR) declined rapidly and markedly after treatment onset, becoming undetectable or detectable at only a low copy number (<10 copies per milliliter) at 4 weeks (23). Monitoring T790M may indicate when the cancer is developing resistance to first- and second-generation TKIs and provides a basis for changing treatment toward drugs that have specific activity on the evolved mutation (34, 36), i.e., third-generation TKIs such as osimertinib or chemotherapy.

Prediction of the treatment outcome requires evaluation of the levels of both sensitizing and resistance mutations. Several studies have shown that in addition to the observation of T790M mutations, an increase in original sensitizing EGFR mutations is associated with progressive disease (PD) diagnosis (61–63). Further, the incidence of PD was almost five times lower in patients without increased levels of sensitizing EGFR mutations in the plasma (64). In addition, the increase or appearance of the plasma T790M allele frequency almost tripled the risk of death or PD (64). Moreover, the increase of sensitizing mutations can occur prior to the detection of resistance mutation of T790M (64). Of 105 patients screened after their progression to EGFR-TKIs, sensitizing mutations and plasma T790M resistance mutation were found in 56.2 and 35.2% of the patients, respectively (15).

Timing is important in cancer treatments. Using liquid biopsies, progression was observed 8 months prior to objective progression, when the concentration of the EGFR mutation increased ≥20% than the lowest concentration recorded during the treatment (65). In addition, early progression as indicated by T790M mutation in plasma can be detected earlier than that detected in computed tomography (CT) scans. In a study of 41 patients, progression was detected from plasma samples 51 days prior to those detected in CT scans (12). Another study that enrolled 102 patients even reported an earlier detection time of 103 days (66).

### Anaplastic Lymphoma Kinase Gene (ALK)

Chromosomal rearrangements in ALK are found in approximately 3–7% of patients with lung cancer (67, 68). Multiple translocation arrangements of the ALK gene have been described in NSCLC, and the most common fusion partner is echinoderm microtubule-associated protein-like 4 (EML4). The presence of ALK translocation is associated with treatment response to ALK-TKI. However, clinical outcomes in these patients vary, and the benefit of TKIs is often short due to acquired resistance. Moreover, multiple secondary ALK mutations have been identified, and novel fusion partners whose sensitivity has not been tested have been identified. One new fusion partner that has been recently reported to be sensitive to crizotinib is GCC2-ALK (35). In majority of cases, ALK translocations do not overlap with other oncogenic mutations found in NSCLC (e.g., EGFR mutations, KRAS mutations, ROS1 gene translocations) (69–72). However, rearrangement of the ALK gene can present as an acquired mutation to EGFR TKI therapy, these fusion mutations and not only the most common resistance mutation T790M should be assessed during EGFR progression (33). ALK translocations can be detected via liquid biopsy using numerous next-generation sequencing (NGS) methods and targeted real-time PCR assays; however, these methods are unlikely to detect fusions with novel partners. Furthermore, detection of mutation variants of ALK may turn out to be useful in selecting the most optimal TKI for therapy, as preclinical results clearly show linkage between mutations and tested TKIs *in vitro*, e.g., in L1196M and S1206Y mutations having resistance to crizotininib but nor ceritinib (20, 73), and central nervous system metastatic ALK+ NSCLC patient responded to lolartinib after ALK G1202R mutation was detected (38). Of course, this needs to be proven in clinical trials.

### ROS Proto-Oncogene 1 (ROS1)

ROS1 gene rearrangements occur in 1–3% of NSCLC (74, 75). ROS1 is a receptor tyrosine kinase of the insulin receptor family, and its rearrangements in NSCLC have been associated with sensitivity to oral ROS1 TKIs. Several ROS1 rearrangements have been described, and the most common fusion partners are CD74, SLC34A2, CCDC6, and FIG. As with ALK, ROS1 translocations can also be detected via liquid biopsy using numerous NGS methods and targeted real-time PCR assays, and fusions with previously known partners can be detected.

### BRAF Proto-Oncogene (BRAF)

BRAF belongs to a family of serine-threonine protein kinases, and it is part of the canonical MAP/ERK signaling pathway. BRAF mutations are found in 1–4% of NSCLC patients, and most of them are adenocarcinomas (76, 77). The presence of a specific mutation resulting in a change in amino acid position 600 (V600E) has been associated with responsiveness to combined therapy with oral inhibitors of BRAF and MEK. In addition, the V600E mutation can develop as a resistance mutation for EGFR-TKI therapy. Although other mutations in BRAF have also identified in NSCLC patients, their importance on therapy selection is currently not well-understood. Liquid biopsy using NGS and targeted real-time PCR assays may identify BRAF mutation status. However, some studies reported that liquid biopsies have lower sensitivity for BRAF than EGFR (78).

### KRAS Proto-Oncogene (KRAS)

KRAS activating mutations develop from unregulated signaling through the MAP/ERK pathway. KRAS is a G-protein with intrinsic GTPase activity. Several different mutations are found in NSCLC, and the most common mutations are in codon 12 (76, 79). Currently, there is no direct anti-KRAS therapy available. However, the detection of KRAS mutation rules out the presence of other actionable driver mutations (80), and thus eliminates the need for further molecular profiling. Moreover, the presence of KRAS mutations is prognostic of poorer survival; it has also been associated with reduced responsiveness to EGFR TKI therapy (29). Thus, the presence of KRAS mutation cannot be used to directly guide therapy decisions. However, evidence suggests that KRAS genotype detected in cfDNA is a negative prognostic factor of survival in NSCLC patients, and the predictive or prognostic role of KRAS mutations in cfDNA remains to be confirmed and warrants further investigation (81).

### Other Gene Mutations

Other less common gene mutations (e.g., AKT, DDR2, FGFR, HER2, MEK1, MET, NTRK1, PI3KCA, PTEN, and RET) have been identified in approximately 1–2% of NSCLC cases. However, there are currently no approved therapies that are available to target these mutations. All of these abnormalities are however detected in the ctDNA isolated from the plasma. In addition, as some of them are potentially treatable, their detection can become clinically important in the future.

### ctDNA and CTC Occurrence

In addition to targeting actionable mutations, liquid biopsy has the potential to track tumor evolution non-invasively with ctDNA occurrence-based methods, which can be easily repeated several times. This may become a useful tool for the diagnosis and treatment of NSCLC. It has been reported that ctDNA can detect early cancers (82), thus creating warranting the need to further assess the clinical validity and utility of this approach. However, complicating factors in early cancer detection are tumor size, tumor necrosis, and proliferation. A recent study by Abbosh et al. (83) concluded that the amount of ctDNA in early stage tumor was under the detection limit owing to its size. Their method had 99% sensitivity to detect single nucleotide variants at frequency above 0.1% (83), creating the need for technological improvements to overcome this problem. Moreover, they found that pre-operative ctDNA was detected in 97% of lung squamous cell carcinoma cases compared to only 19% of adenocarcinoma (83), indicating that not only the size of the tumor, but also the histological subtype is a biological factor for the accuracy of liquid biopsies. These aspects need to be addressed before liquid biopsy can be used as a routine screening modality in NSCLC.

In addition, the appropriate amount of ctDNA that can be used as a predictive marker for prognosis and treatment response is unclear because changes in ctDNA mutations have been observed in as early as 2 days after surgery for lung cancer (84). The overall ctDNA concentration has also been used as a follow-up marker for early detection of relapse following primary surgery (83). The persistent detection of postoperative ctDNA predicted relapse in 93% of patients with NSCLC, with a median lead time of 60 days prior to radiological confirmation (83). In other cancer types, the persistent detection of ctDNA after local therapy (surgery or radical radiotherapy) was a predictive factor for a higher risk of relapse in some proof of concept studies [colon cancer: (85, 86); breast cancer: (87, 88); and pancreatic cancer: (89)]. However, the total concentration of cfDNA may not predict chemotherapy response (90). Meanwhile, monitoring of the disease progression based on the amount of ctDNA mutations was shown to be more reliable than measuring the diameter of the target tumor lesions (65), and the presence of ctDNA had a higher positive predictive value than that of six tumor biomarkers currently used (CA125, CA19-9, CYFRA21-1, CEA, NSE, and squamous cell carcinoma antigen) (84). In addition to ctDNA appearance before and after treatment, the presence of CTCs has been repeatedly reported to be a good predictor of disease progression (35, 91). In a study of 102 patients, postoperative CTCs were related to shorter recurrence-free survival (92).

## Liquid Biopsies During Disease Development

Despite the emerging diagnostic tools and therapeutic advances for cancer, lung cancer still remains the leading cause of cancer-related mortality (1). Lung cancer is often diagnosed at an advanced stage due to inadequate screening methods, the late onset of clinical symptoms, and the late referral of patients to examinations. Increasing knowledge on the molecular biomarkers and their relationship with cancer cell growth, targeted therapies, and survival will have a huge clinical impact. Some studies have suggested the potential of liquid biopsies for early detection of NSCLC (82, 83). However, whether liquid biopsies is a reliable modality for the early detection of cancer in asymptomatic individuals and populations is unclear. Currently, liquid biopsies are not used for the early detection or screening of NSCLC.

The NCCN recommends that all patients, except those with squamous cell lung cancer who have a smoking history, should be screened for the presence of activating EGFR mutations, i.e., deletions in exon 19 or point mutation L858R in exon 21, to identify those who will benefit from EGFR TKI treatment. Some studies supported the appropriateness of liquid biopsy for guiding the therapy decision. In a large cohort study of 1,026 patients, liquid biopsy was useful for guiding EGFR TKI treatment in 11% of patients (15). The use of liquid biopsy for guiding EGFR TKI treatment resulted in improved progression-free survival (PFS) compared with that in standard chemotherapy (50, 51). Similar results in patients with ALK and ROS1 rearrangements have been achieved. First-line therapy with crizotinib based on liquid biopsy findings improved PFS, and the response rate in ALK-positive NSCLC patients (93) was approximately 70%, including complete responses for patients with ROS1 mutations (74). Thus, liquid biopsies are equally important to guide targeted therapies in patients with ALK and ROS1 rearrangements (94). In addition, it is important to identify KRAS-positive patients, who account for as high 25% in the North American population (95, 96), who do not benefit from EGFR TKIs and where no targeted therapy is currently available.

**Figure 1:**
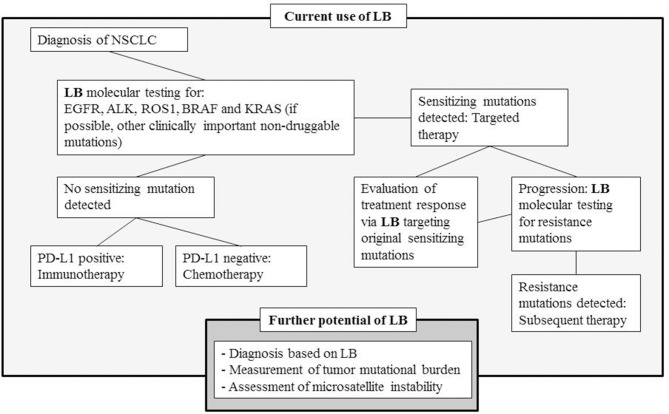
Simplified treatment diagram of NSCLC and the role of liquid biopsies at the different phases of disease development, and the further potential of LB in the evolving field of personalized medication (LB, liquid biopsy; EGFR, epidermal growth factor receptor; ALK, anaplastic lymphoma kinase; ROS1, ROS proto-oncogene 1; BRAF, BRAF proto-oncogene; KRAS, KRAS proto-oncogene).

In addition to initial molecular diagnosis and targeted therapy decisions, liquid biopsies have shown their potential as a follow-up modality evaluating treatment response and detecting progression during TKI treatment (31). Acquired resistance to targeted inhibitors is nearly universal as more than 50% of patients treated with first- and second-generation EGFR TKI harbor T790M mutation, and one-third of patients treated with the first-generation ALK TKI crizotinib acquire resistance due to various ALK-specific point mutations that interfere with drug binding. Liquid biopsies enable longitudinal binding that allows for the follow-up of both the changes in the original sensitizing mutations and the development of resistance mutations. The different applications of liquid biopsy in NSCLC are presented in Figure 1.

We acknowledge the wide molecular landscape of NSCLC and all lung cancers; thus, we have not been able to cover this area thoroughly. In addition, immune-oncology is an expanding field; particularly, the use of immunotherapies in NSCLC is increasing. Liquid biopsies, particularly CTCs, have been used to evaluate patients with PD-L1 expression (97), and a positive expression of which has been associated with poor prognosis [(98, 99). In addition, immune-oncology appears to focus on the assessment of mutational burden and microsatellite instability from liquid biopsy samples.

## Discussion

No reliable biomarker that can be used for patient monitoring during treatments and to modify treatments accordingly has been previously identified in lung cancer. Although albumin has been used to determine the patient's general condition (100), and carcinoembryonic antigen (CEA) has been correlated with adenocarcinoma cancers (101) and neuron-specific enolase (NSE) in epithelial cancers (102), they cannot be used reliably for treatment decisions. Fortunately, various potentially actionable alterations that are detectable in patients with NSCLC have now been identified. In addition, as the acquired resistance to targeted inhibitors is nearly universal, the development of targeted therapies and molecular identification is rapidly evolving. Thus, treatment decision making is becoming more individualized owing to molecular testing and liquid biopsy.

Liquid biopsies have been successfully used to guide treatment decisions in patients with positive EGFR and ALK mutations; the response rates and PFS were similar compared with treatments decided made based on molecular analysis of tissue biopsy (15, 31, 32). However, studies showing that treatment decisions based on liquid biopsies result in better outcomes in patients with other treatable mutations are limited. Further studies are needed to firmly establish the usefulness of liquid biopsy for detecting molecular markers in clinical practice. In addition, the presence of ctDNA and CTC before and after lung cancer surgery should be studied further to determine whether they indicate the need for adjuvant therapy regardless of the tumor size or nodal status. Currently, one of the most promising use of liquid biopsies is in the detection of cancer progression and development of drug resistance. T790M mutation is currently used as an indicator of treatment efficacy in patients treated with EGFR TKI, and it has shown promising potential. Based on the Darwinian clonal evolution as well the need for therapies targeting only part of the tumor cell clones, the genomic composition of individual tumor changes over time, leading to resistance. In lung cancer, the use of TKIs frequently results in resistant cell clones and loss of responsiveness in tumors. This has led to the development of an increasing number of new molecular analysis methods and fourth-generation TKIs. In addition, the treatment guidelines for NSCLC are constantly changed according to the newest clinical findings. Accordingly, guidelines for liquid biopsies need to be continuously adjusted to suit the current clinical needs. For example, if the third-generation TKI osimertinib will be used as first-line treatment, then the detection of T790M mutation might have less clinical importance.

The high interest on liquid biopsies is evident in the rapid increase in the use of liquid biopsy in clinical trials, comparative molecular studies, and published studies. Information obtained from blood-based liquid biopsy (B) has been suggested to be included in the TNM staging system, which is generally referred to as the TNMB system (78), and new statements and guidelines are published as the knowledge increases [(20, 103–105). According the statement paper of International Association for the Study of Lung Cancer (IASLC), a positive EGFR, ALK, ROS1, or BRAF result of liquid biopsy NGS should be considered adequate to initiate first-line therapy in advanced NSCLC, however, a negative results requires a confirmation from tumor biopsy (20). In addition, the IASLC recommends that treatable mutations should not be investigated if there are no local drugs available for such mutations (20). However, we disagree with such recommendation because although access to drugs used in targeted therapies can be limited, there are possible replacements. For example, genetic findings, such as T790M mutations, can be used as basis to switch treatment to chemotherapy if osimertinib is not available.

Various factors are guiding treatment decision, including tumor type, grade, and stage; genetic findings; patient's performance status; and prior therapies. In addition, similar variants may have different therapeutic consequences, depending on the site of the primary tumor and individual behavior of the cancer. The action of a particular genomic variant should be cautiously stated in a ctDNA report. Liquid biopsy is yet to be used as a standard modality for cancer diagnosis or assessment of treatment response, and paraffin block analysis or medical imaging remains the standard modalities for such purposes. However, ctDNA assay provides critical new information that can be used in patient management and allows for the monitoring of signs of treatment resistance by providing data on emerging clones. Clearly, further research is needed before liquid biopsy can be successfully applied in routine clinical practice. Moreover, the stage at which second-line treatments should be started and at which the treatment should be changed and the real significance of liquid biopsy in patient outcomes compared with patient management guided via molecular data and imaging modalities need to be addressed. This review showed that liquid biopsies now play a critical role in EGFR sensitizing and resistance mutations. However, in other known and targetable alterations, prospective evidence on the use of liquid biopsy to achieve the best benefit for NSCLC patients is still needed.

## Author Contributions

JS conducted the PubMed search. NE conducted the clinicaltrial.gov search. All authors provided critical feedback and helped to shape the manuscript. JS took the lead in writing the manuscript with help of all authors on contributing to the final manuscript.

### Conflict of Interest Statement

The authors declare that the research was conducted in the absence of any commercial or financial relationships that could be construed as a potential conflict of interest.
