# Supplementary material for: The Value of Liquid Biopsies for Guiding Therapy Decisions in Non-small Cell Lung Cancer
Source: Front Oncol. 2019 Mar 5;9:129. doi: 10.3389/fonc.2019.00129 (PMC6411700; doi:10.3389/fonc.2019.00129)
Supplement: Supplementary file 1 [file Data_Sheet_1.PDF]

Supplementary material

The value of liquid biopsies for guiding therapy decisions in non-small cell lung cancer

Jatta Saarenheimo[1,2*], Natalja Eigeliene[3,4], Heidi Andersen[5], Marja Tiirola[2] and Antti Jekunen[3,4]

1 Department of Pathology, Vasa Central Hospital, Finland
2 University of Jyväskylä, Department of Biological and Environmental Science
3 Department of Oncology, Vasa Central Hospital, Finland
4 University of Turku, Department of Oncology and Radiotherapy
5 Department of Pulmonology, Vasa Central Hospital, Finland

* corresponding author: Jatta Saarenheimo, PhD
Department of Pathology, Vasa Central Hospital, Hietalahdenkatu 2-4, 65130, Vaasa, Finland
Phone: +358-6-213 1111, E-mail: jatta.saarenheimo@vshp.fi

Supplementary Table 1. Number of peer reviewed publications in PubMed with search words; "cancer", "non-small cell lung cancer (NSCLC)", and "liquid biopsy" . Search done 1.9.2018.

Year	Cancer	NSCLC	NSCLC liquid biopsy	% of liquid biopsy publications of NSCLC
**2010**	116406	3065	3	0.10
**2011**	124473	3301	1	0.03
**2012**	137091	4020	5	0.12
**2013**	145613	4460	2	0.04
**2014**	159727	4889	6	0.12
**2015**	169686	5558	18	0.32
**2016**	171636	5411	43	0.79
**2017**	171914	5678	101	1.78
**2018**	125991	4266	68	1.60

Supplementary Table 2. Distributions of published articles topics from PubMed with search words; "NSCLC liquid biopsy". Search done 1.9.2018.

	**Number of papers**	**% of all papers**
All papers	247	
Reviews	78	32%
Clinical trials	8	3%
Case reports	10	4%

Supplementary Table 3. Number and distribution of active, recruiting and completed clinical trials from clinicaltrials.gov search. Search done 12.5.2018

**Search words**	**All studies**	**Clinical trials**	**Active**	**Recruiting**	**Completed**
lung cancer, plasma	602	209	84	198	187
lung cancer, liquid biopsies	36	11	11	11	1
lung cancer, CTCs	708	657	298	217	199
lung cancer, cell free DNA	24	12	2	6	2
lung cancer, circulating tumor DNA	79	38	11	42	7
lung cancer, exosomes	8	5	3	3	1
